# Cell differentiation versus cell death: extracellular glucose is a key determinant of cell fate following oxidative stress exposure

**DOI:** 10.1038/cddis.2014.52

**Published:** 2014-02-20

**Authors:** R C Poulsen, H J Knowles, A J Carr, P A Hulley

**Affiliations:** 1 Botnar Research Centre, University of Oxford, Oxford, UK

**Keywords:** hyperglycaemia, sirtuin 3, hypoxia-inducible factor, forkhead, tenocyte, type 1 collagen

## Abstract

Cells, particularly mechano-sensitive musculoskeletal cells such as tenocytes, routinely encounter oxidative stress. Oxidative stress can not only stimulate tissue repair, but also cause damage leading to tissue degeneration. As diabetes is associated with increased oxidative damage as well as increased risk of tendon degeneration, the aim of this study was to determine if extracellular glucose levels alter the response of tendon cells to oxidative stress. Primary human tenocytes were cultured in either high (17.5 mM) or low (5 mM) glucose and treated with 100 *μ*M hydrogen peroxide. In low glucose, peroxide-treated cells remained fully viable and collagen synthesis was increased, suggesting an anabolic response. In high glucose, however, peroxide treatment led to increased bim-mediated apoptosis. The activities of both forkhead box O (FOXO1) and p53 were required for upregulation of *bim* RNA expression in high glucose. We found that both p53-mediated inhibition of the bim repressor micro RNA (miR17-92) and FOXO1-mediated upregulation of *bim* transcription were required to permit accumulation of *bim* RNA. High glucose coupled with oxidative stress resulted in upregulation of miR28-5p, which directly inhibited expression of the p53 deacetylase sirtuin 3, resulting in increased levels of acetylated p53. In peroxide-treated cells in both high and low glucose, protein levels of acetylated FOXO1 as well as HIF1*α* (hypoxia-inducible factor 1*α*) were increased. However, under low-glucose conditions, peroxide treatment resulted in activation of p38, which inhibited FOXO1-mediated but promoted HIF1*α*-mediated transcriptional activity. In low glucose, HIF1*α* upregulated expression of sox9 and scleraxis, two critical transcription factors involved in establishing the tenocyte phenotype, and increased collagen synthesis. The switch from FOXO1-mediated (proapoptosis) to HIF1*α*-mediated (prodifferentiation) transcription occurred at an extracellular glucose concentration of 7 mM, a concentration equivalent to the maximum normal blood glucose concentration. Extracellular glucose has a profound effect on the cellular response to oxidative stress. A level of oxidative stress normally anabolic may be pathological in high glucose.

Oxidative stress is both beneficial and harmful in biological systems. Generated during aerobic metabolism and in response to mechanical loading, reactive oxygen species (ROS) are critical secondary messengers essential for the promotion of cell differentiation and tissue remodelling.^1, 2^ However, damage caused by oxidative stress is a major contributor to tissue degeneration and disease.^2, 3^ Cells have developed a number of strategies to minimize ROS-induced damage. Transcription factors such as p53 and the forkhead box O (FOXO) are induced by oxidative stress and directly regulate expression of genes whose products mediate antioxidant defence and damage repair.^4, 5, 6^ In the event of irreparable ROS-mediated damage, p53 and FOXOs can induce apoptosis,^7, 8^ thereby sacrificing the afflicted cell to avoid compromising overall tissue health.

Although oxidative stress promotes FOXO and p53 expression, metabolic cues fine-tune their activity. Modulators of FOXO and p53 activity include phosphatidylinositol-3-kinase (PI3K), the sirtuins, and p38 mitogen-activated protein kinase (MAPK), all of which are regulated by cellular energy status.^7, 9, 10, 11^ Metabolic sensors can also regulate the prosynthetic response of cells to oxidative stress as PI3K, the sirtuins, and p38 also regulate activity of hypoxia-inducible factor 1*α* (HIF1*α*),^12, 13, 14^ a transcription factor that can be induced by oxidative stress under normoxic conditions^15^ and that promotes collagen deposition.^16, 17^ As a result, PI3K, sirtuins, and p38 can have powerful roles in controlling which of the transcription factors induced in response to oxidative stress are activated.

Diabetes and hyperglycaemia are risk factors for the development of a number of degenerative conditions, including those affecting musculoskeletal tissues.^18, 19, 20, 21^ The tendon appears to be particularly susceptible to damage with one study reporting degenerative changes in ∼ 40% of diabetic patients.^20^ Oxidative damage and excessive cell death are also common features of degenerative conditions,^3, 22^ raising the possibility that the ability of tissues to withstand oxidative stress may be impaired by hyperglycaemia. The purpose of this study was to determine whether extracellular glucose levels influence the cellular response to oxidative stress, a stressor to which cells are frequently exposed.

## Results

### Extracellular glucose controls cell fate following oxidative stress

**Figure 1:**
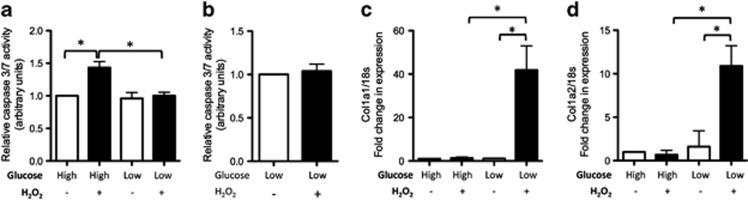
Exposure to oxidative stress results in different cell fate outcomes depending on the level of extracellular glucose (**a**) Level of apoptosis, as determined by measuring caspase 3/7 activity, was significantly higher in peroxide-treated cells cultured in high (17.5 mM) glucose compared with either untreated controls or peroxide-treated cells cultured in low (5 mM) glucose following 18 h of treatment. (**b**) No increase in level of apoptosis occurred in peroxide-treated cells cultured in low glucose for 7 days. RNA levels of (**c**) *col1a1* and (**d**) *col1a2* were significantly higher in peroxide-treated cells cultured in low glucose compared with untreated controls or peroxide-treated cells cultured in high glucose. Statistically significant (*P*≤0.05) differences between treatments are indicated by *. Experiments were performed on cells isolated from a minimum of three different patients. Results are expressed as mean±S.D.

Primary human tenocytes were treated with varying concentrations of hydrogen peroxide to determine the concentration that is proapoptotic to these cells. A significant increase in the level of apoptosis was evident in cells treated with 100 *μ*M peroxide but not in cells treated with lower peroxide concentrations (Supplementary Figure). In order to determine if extracellular glucose levels influence cell fate following exposure to oxidative stress, tenocytes were cultured in either standard media (containing 17.5 mM glucose, ‘high glucose') or media containing 5 mM glucose (‘low glucose') and treated with 100 *μ*M H_2_O_2_ for 18 h. Level of apoptosis (determined by measuring caspase 3/7 activity) was ∼ 1.5 times greater in peroxide-treated cells cultured in high glucose compared with untreated controls (Figure 1a). Level of apoptosis was not increased in peroxide-treated cells cultured in low glucose following 18 h (Figure 1a) or 7 days (Figure 1b) of treatment. Compared with untreated controls, peroxide-treated cells cultured in low glucose expressed higher RNA levels of col1a1 and col1a2, the gene products of which form type 1 collagen (Figures 1c and d), suggesting a level of oxidative stress lethal to cells in high glucose may be anabolic to cells in low glucose.

### Both p53 and FOXO1 are required for apoptosis in oxidative stress-exposed cells cultured in high glucose

**Figure 2:**
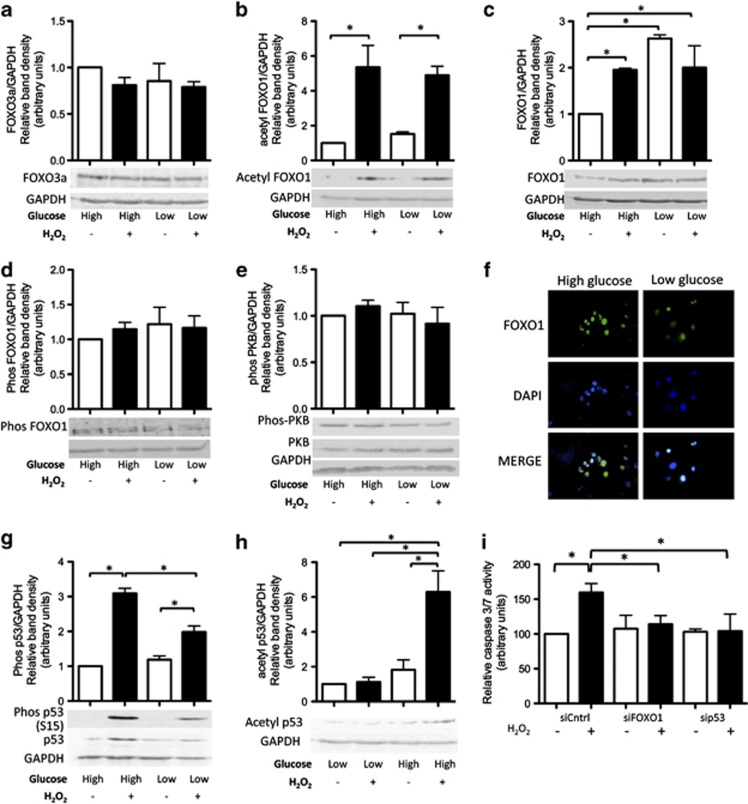
FOXO1 and p53 are activated in oxidative stress-exposed cells in high glucose (**a**) Protein levels of FOXO3a quantified by measuring relative band density from western blots were unchanged by peroxide treatment. (**b**) In contrast, levels of acetylated FOXO1 were significantly higher in peroxide-treated cells in both high and low glucose. (**c**) Protein levels of total FOXO1 were significantly higher in peroxide-treated cells cultured in high glucose compared with untreated controls. FOXO1 protein levels were also significantly higher in cells cultured in low compared with high glucose. There was no significant difference in FOXO1 protein levels between peroxide-treated or untreated cells cultured in low glucose. No change in levels of (**d**) phosphorylated PKB or (**e**) phosphorylated FOXO1 was observed. (**f**) By immunocytochemistry using a FOXO1-targeting primary antibody and a dylight-488 (green) secondary antibody, FOXO1 was found to be present in the nucleus in peroxide-treated cells in both high and low glucose. DAPI (blue) was used to label nuclei. Levels of (**g**) phosphorylated p53 (S15) and (**h**) acetylated p53 (K382) were significantly higher in peroxide-treated cells compared with untreated controls. Although phosphorylated p53 was detected in peroxide-treated cells in low glucose, levels were lower than those in peroxide-treated cells in high glucose. No increase in level of acetylated p53 was apparent in peroxide-treated cells in low glucose. (**i**) Level of apoptosis (as measured by caspase 3/7 activity) was not significantly different from untreated controls in peroxide-treated cells in which either FOXO1 or p53 expression had been knocked down by RNAi. Refer Supplementary Information for western blots demonstrating level of knockdown achieved by RNAi. Statistically significant (*P*≤0.05) differences between treatments are indicated by *. Results are expressed as mean±S.D. Experiments were performed on cells isolated from a minimum of three different patients. Representative western blotting and photomicrograph images are shown

As the FOXO forkheads and p53 are known to induce apoptosis in oxidative stress-exposed cells, we wanted to determine if there was any difference in expression of these transcription factors in peroxide-treated cells in high compared with low glucose. By western blotting, we found that the protein levels of FOXO3a were unchanged (Figure 2a); however, levels of acetylated FOXO1 (Figure 2b) were markedly higher in peroxide-treated cells compared with untreated controls regardless of the level of extracellular glucose. Levels of total FOXO1 (Figure 2c) were higher in peroxide-treated cells in high glucose compared with untreated controls; however, the difference was less dramatic than that seen for acetylated FOXO1 (Figure 2b). Interestingly, basal levels of FOXO1 expression were higher in cells cultured in low glucose compared with high glucose. No additional increase in FOXO1 protein levels was apparent following peroxide treatment of cells in low glucose (Figure 2c). There was no difference in level of phosphorylated protein kinase B (PKB) (Figure 2d) or in levels of FOXO1 phosphorylated on the PKB-target residue S256 (Figure 2e) in peroxide-treated cells cultured in high compared with low glucose. Using immunocytochemistry, we found FOXO1 was predominately localized in the nucleus in peroxide-treated cells cultured in high as well as low glucose (Figure 2f).

Levels of phosphorylated (S15) p53 were significantly higher in peroxide-treated cells cultured in high glucose compared with either untreated controls or peroxide-treated cells cultured in low glucose (Figure 2g). Levels of p53 acetylated on K382 were significantly greater in peroxide-treated cells cultured only in high, not low glucose (Figure 2h).

To determine the roles of FOXO1 and p53 in peroxide-treated cells cultured in high glucose, we used RNA interference (RNAi) to knock down FOXO1 or p53 expression (Supplementary Figure). Knockdown of either FOXO1 or p53 completely prevented apoptosis in peroxide-treated cells cultured in high glucose (Figure 2i), indicating that the presence of both transcription factors is required for apoptosis.

**Figure 3:**
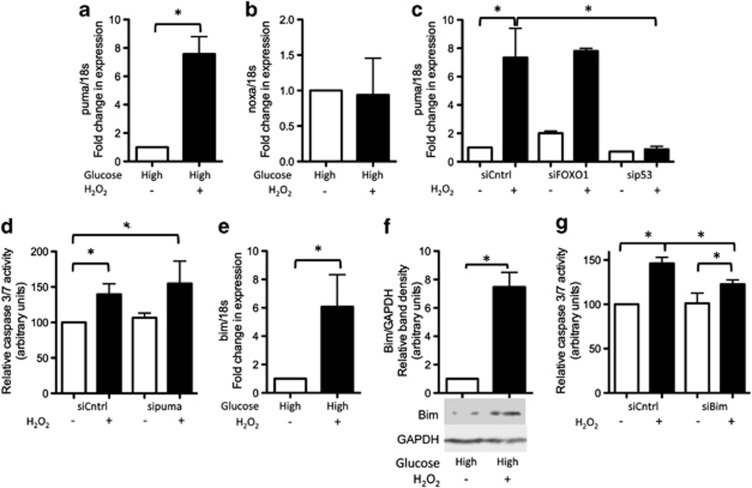
Oxidative stress-induced apoptosis in cells cultured in high glucose is mediated by bim RNA levels of (**a**) *puma* but not (**b**) *noxa* were significantly higher in peroxide-treated cells cultured in high glucose compared with untreated controls. No increase in (**c**) RNA levels of *puma* were apparent in peroxide-treated cells in which p53 (but not FOXO1) expression had been knocked down by RNAi. (**d**) There was no difference in level of apoptosis in peroxide-treated cells in which *puma* expression had been knocked down by RNAi compared with peroxide-treated controls. (Refer Supplementary Information for western blots demonstrating level of knockdown achieved by RNAi.) Both (**e**) RNA and (**f**) protein levels of bim were higher in peroxide-treated cells cultured in high glucose compared with untreated controls. (**g**) Level of apoptosis was significantly lower in peroxide-treated cells in which bim had been knocked down by RNAi compared with peroxide-treated controls. (Refer Supplementary Information for western blots demonstrating level of knockdown achieved by RNAi.) Statistically significant (*P*≤0.05) differences between treatments are indicated by *. Results are expressed as mean±S.D. Experiments were performed on cells isolated from a minimum of three different patients. Western blot images shown are representative of those obtained for all patients

Both FOXO1 and p53 can directly regulate transcription of genes such as *puma* and *noxa* whose products are involved in apoptosis induction.^23, 24^ We found that the RNA levels of *puma*, but not *noxa*, were significantly higher in oxidative stress-exposed cells cultured in high glucose (Figures 3a and b). *Puma* RNA levels were significantly lower in peroxide-treated cells cultured in high glucose in which p53 (but not FOXO1) had been knocked down by RNAi, indicating that p53 activity was largely responsible for driving the increase in *puma* levels under these conditions (Figure 3c). However, knockdown of *puma* had no significant effect on the level of apoptosis following peroxide treatment, suggesting that under the experimental conditions employed loss of puma activity was insufficient to prevent apoptosis induction (Figure 3d, Supplementary Figure).

Bim is another proapoptotic protein known to be involved in oxidative stress-induced apoptosis.^25^ We found that RNA (Figure 3e) and protein (Figure 3f) levels of bim were elevated in peroxide-treated cells cultured in high glucose. Level of apoptosis was significantly lower in peroxide-treated cells in which *bim* expression had been knocked down by RNAi (Supplementary Figure), indicating that bim had a major role in apoptosis initiation (Figure 3g).

### p53 cooperates with FOXO1 to increase *bim* RNA levels by inhibiting expression of miR17-92

**Figure 4:**
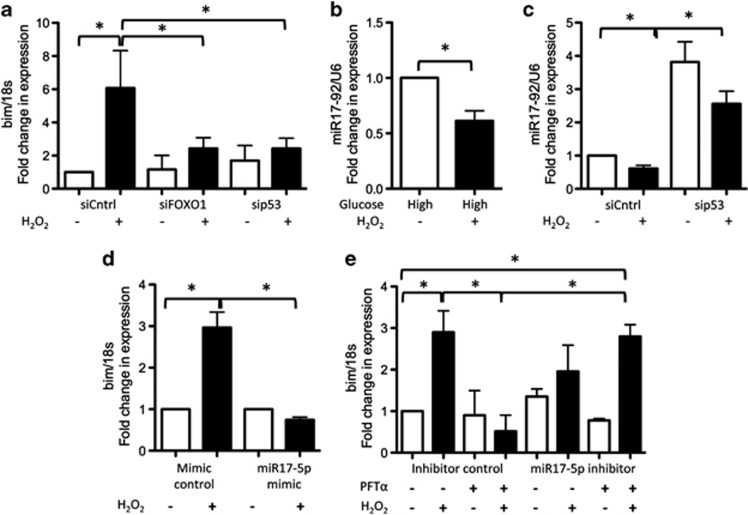
FOXO1 and p53 cooperate to upregulate bim expression. (**a**) RNA levels of *bim* were lower in peroxide-treated cells in which expression of either FOXO1 or p53 had been knocked down by RNAi. (**b**) Levels of the miRNA cluster miR17-92 were significantly lower in peroxide-treated cells cultured in high glucose compared with untreated controls. (**c**) miR17-92 levels were significantly higher in cells in which p53 expression had been knocked down by RNAi compared with controls. (**d**) RNA levels of *bim* were significantly lower in peroxide-treated cells cultured in high glucose transfected with a miR17-5p mimic compared with peroxide-treated controls. (**e**) RNA levels of *bim* were significantly lower in peroxide-treated cells cultured in high glucose cotreated with the p53 inhibitor PFT*α* compared with cells treated with peroxide alone or compared with cells cotreated with peroxide and PFT*α* and transfected with a miR17-5p inhibitor. Statistically significant (*P*≤0.05) differences between treatments are indicated by *. Experiments were performed on cells isolated from a minimum of three different patients

Surprisingly, we found that RNA expression of *bim* was significantly lower in peroxide-treated cells in which either FOXO1 or p53 expression had been knocked down compared with peroxide-treated controls (Figure 4a). Although FOXO1 is known to regulate *bim* transcription,^23^ *bim* is not a transcriptional target of p53. p53 is known to inhibit the expression of micro RNA (miR17-92),^26^ a cluster of miRNAs that includes the negative regulator of *bim* miR17-5p.^27^ We found that levels of miR17-92 were significantly lower in peroxide-treated cells cultured in high glucose compared with untreated controls (Figure 4b) or compared with cells in which p53 expression had been knocked down (Figure 4c). Using an miR17-5p mimic, we overexpressed miR17-5p in tenocytes. We found that RNA levels of *bim* were significantly lower in peroxide-treated cells cultured in high glucose expressing the mimic compared with peroxide-treated controls (Figure 4d). Next, we transfected cells with an miR17-5p inhibitor before treatment with the p53 inhibitor pifithrin-*α* (PFT*α*). In cells expressing the miR17-5p inhibitor, RNA levels of *bim* were significantly higher following cotreatment with peroxide and PFT*α* compared with cells expressing a nontargeting miR inhibitor control (Figure 4e). These results demonstrate that inhibition of miR17-5p can compensate for loss of p53 activity in peroxide-treated cells and restore *bim* expression.

### Reduced sirtuin 3 expression in peroxide-treated cells cultured in high glucose contributes to an increase in acetylated p53 levels

**Figure 5:**
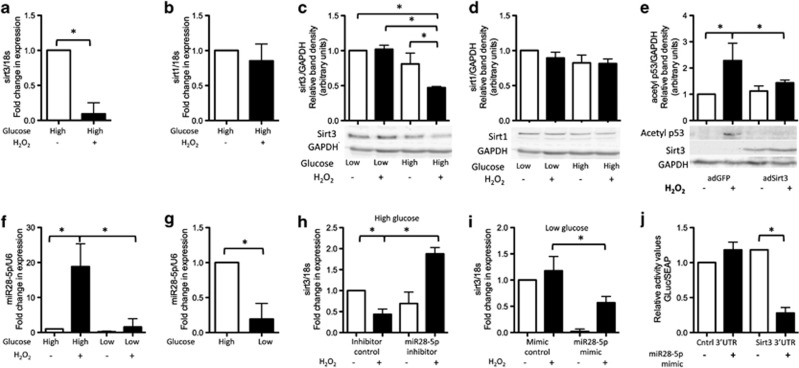
Sirtuin 3 is a novel target of miR28-5p. RNA levels of (**a**) *sirtuin 3* but not (**b**) *sirtuin 1* were lower in peroxide-treated cells cultured in high glucose compared with untreated controls. Similarly, protein levels (as measured by relative quantification of the band density from western blots) of (**c**) sirtuin 3 were significantly lower in peroxide-treated cells cultured in high glucose compared with untreated controls or cells cultured in low glucose with or without peroxide treatment. (**d**) However, protein levels of sirtuin 1 were not significantly different between treatments. (**e**) No increase in the level of acetylated p53 was observed in peroxide-treated cells cultured in high glucose and infected with a sirtuin 3-bearing adenoviral vector (adSirt3). A GFP-bearing adenoviral vector (adGFP) was used as a control. (**f**) Levels of miR28-5p were significantly higher in peroxide-treated cells cultured in high but not low glucose compared with untreated controls. (**g**) Graph highlighting the difference in levels of miR28-5p in untreated cells in high and low glucose (note the *y*-axis scale differs from that in **f**). Levels of miR28-5p were higher in untreated cells cultured in high glucose compared with in cells cultured in low glucose. (**h**) In cells cultured in high glucose, RNA levels of sirtuin 3 were significantly higher in peroxide-treated cells transfected with a miR28-5p inhibitor compared with peroxide-treated controls. (**i**) Conversely, in cells cultured in low glucose, RNA levels of sirtuin 3 were significantly lower in cells expressing a miR28-5p mimic compared with peroxide-treated controls. (**j**) Activity of a 3′-UTR sirtuin 3 reporter in which the 3′ UTR sequence of sirtuin 3 had been inserted downstream of the secreted GLuc reporter gene was significantly lower in cells transfected with a miR28-5p mimic compared with non-mimic expressing cells. A secreted alkaline phosphatase (SEAP) reporter driven by the CMV promoter cloned into the same vector served as an internal control for transfection efficiency. The miR28-5p mimic had no effect on the negative control 3′UTR construct, demonstrating its specificity for the sirtuin 3 3′-UTR. Statistically significant (*P*≤0.05) differences between treatments are indicated by *. Results are expressed as mean±S.D. Experiments were performed on cells isolated from a minimum of three different patients. Western blot images shown are representative of those obtained for all patients

Next, we wanted to understand why an increase in levels of acetylated p53 only occurred in peroxide-treated cells cultured in high but not low glucose. Two members of the sirtuin family, sirtuins 1 and 3, are known to deacetylate p53.^28, 29^ We found RNA (Figures 5a and b) and protein (Figures 5c and d) levels of sirtuin 3 but not sirtuin 1 were significantly lower in peroxide-treated cells cultured in high (but not low) glucose compared with untreated controls. To determine whether the reduced levels of sirtuin 3 were responsible for the increased levels of acetylated p53 in peroxide-treated cells, we overexpressed sirtuin 3 in these cells using an adenoviral vector bearing a sirtuin 3 construct (adSirt3). We found that the levels of acetylated p53 were lower in adSirt3-infected peroxide-treated cells compared with peroxide-treated cells infected with a green fluorescent protein (GFP)-bearing adenoviral control (adGFP) (Figure 5e). These results indicate that the reduction in sirtuin 3 levels in peroxide-treated cells contributes to the increase in levels of acetylated p53 in these cells.

### Sirtuin 3 is a direct target of miR28-5p

To determine the mechanism responsible for the reduction in sirtuin 3 levels in peroxide-treated cells in high glucose, we performed a database search of miRNAs predicted to target *sirtuin 3*. We found that the levels of one candidate miRNA, miR28-5p, were significantly higher in peroxide-treated cells cultured in high but not low glucose compared with untreated controls (Figure 5f). Interestingly, levels of miR28-5p were markedly higher in untreated controls cultured in high compared with low glucose, suggesting that extracellular glucose levels may regulate miR28-5p expression (Figure 5g).

To determine if miR28-5p inhibits *sirtuin 3* expression, we transfected peroxide-treated cells cultured in high glucose with a miR28-5p inhibitor. We found that RNA levels of *sirtuin 3* were significantly higher in inhibitor-treated cells compared with controls (Figure 5h). Next, we transfected peroxide-treated cells cultured in low glucose with a miR28-5p mimic and found that levels of *sirtuin 3* were significantly lower in mimic-treated cells compared with controls (Figure 5i). To confirm if *sirtuin 3* is a direct target of miR28-5p, we simultaneously transfected cells with the miR28-5p mimic and a reporter plasmid in which the 3′ UTR sequence of sirtuin 3 had been inserted downstream of the secreted Gaussia luciferase (GLuc) reporter gene driven by a SV40 promoter. We found that activity of the GLuc-sirt3 3′UTR reporter was significantly lower in cells transfected with the miR28-5p mimic compared with cells transfected with a mimic control (Figure 5j). These data indicate that upregulation of miR28-5p in peroxide-treated cells cultured in high glucose results in inhibition of *sirtuin 3* expression.

### p38*α* is activated in oxidative stress-exposed cells under low glucose and promotes HIF1*α* activity

**Figure 6:**
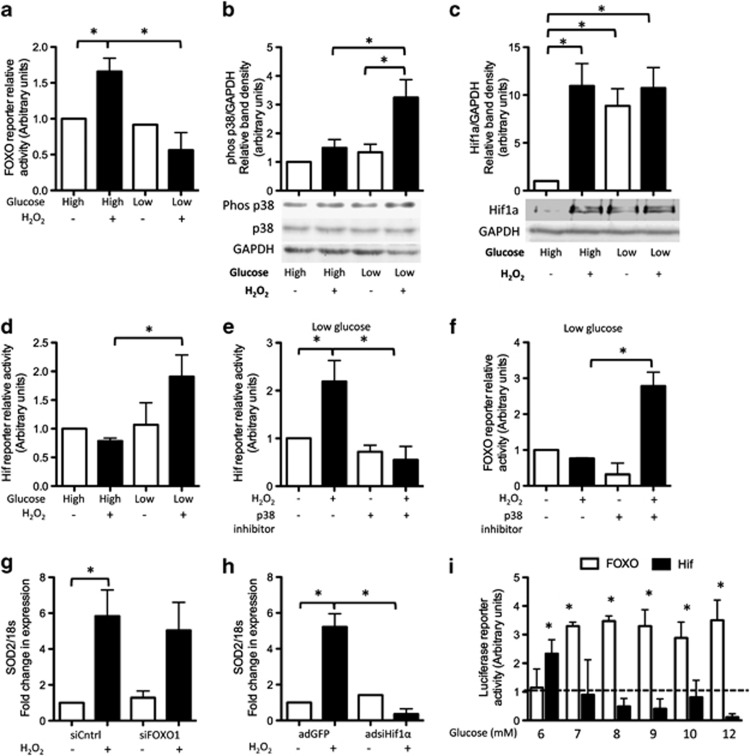
Transcriptional activity of FOXO1 and HIF1*α* is governed by p38. (**a**) Activity of a FOXO luciferase reporter was significantly higher in peroxide-treated cells cultured in high but not low glucose compared with untreated controls. (**b**) Levels of phosphorylated p38 (as determined by western blot) were higher in peroxide-treated cells cultured in low but not high glucose compared with untreated controls. (**c**) Protein levels of HIF1*α* were higher in peroxide-treated cells cultured in either high or low glucose. Protein levels of HIF1*α* were also higher in untreated cells cultured in low glucose compared with untreated cells cultured in high glucose. (**d**) Activity of a HIF luciferase reporter was significantly higher than untreated controls in peroxide-treated cells cultured in low but not high glucose. (**e**) In low glucose, activity of the HIF reporter was significantly lower in peroxide-treated cells cotreated with a p38 inhibitor (SB202190, 10 nM) compared with cells treated with peroxide alone. (**f**) Activity of the FOXO luciferase reporter was significantly higher in peroxide-treated cells in low glucose cotreated with the p38 inhibitor compared with non-inhibitor-treated cells. (**g**) RNA levels of *SOD2* were significantly higher in peroxide-treated cells in low glucose compared with untreated controls. Knockdown of FOXO1 by RNAi had no effect on *SOD2* RNA expression. However, (**h**) *SOD2* RNA levels were significantly lower in peroxide-treated cells in which Hif1*α* expression had been knocked down by RNAi. Statistically significant (*P*≤0.05) differences between treatments are indicated by *. (**i**) Activity of the HIF luciferase reporter (black bars) and FOXO luciferase reporter (white bars) in peroxide-treated cells cultured in varying concentrations of glucose compared with non-peroxide-treated cells (assigned the arbitrary value of 1 and shown by the dashed line). There was no difference in FOXO or HIF reporter activity between non-peroxide-treated cells cultured in different concentrations of glucose. Statistically significant (*P*≤0.05) differences in either FOXO or HIF reporter activity between peroxide-treated cells and untreated controls for each glucose concentration are indicated by *. Results are expressed as mean±S.D. All experiments were performed on cells isolated from at least three different patients. Images shown are representative of those obtained for all patients

Although levels of acetylated p53 were not increased in peroxide-treated cells cultured in low glucose, levels of acetylated FOXO1 were still elevated. To determine why FOXO1 did not induce apoptosis in peroxide-treated tenocytes cultured in low glucose, we measured transcriptional activity of FOXO1 using a FOXO luciferase reporter. We found that the reporter activity was significantly higher in peroxide-treated cells cultured in high but not low glucose, indicating that FOXO1 was not transcriptionally active in low glucose (Figure 6a). p38 has previously been shown to act as a molecular switch inhibiting FOXO-dependent transcription and promoting that of HIF1*α*.^14^ We found that phosphorylated p38*α* levels were higher in peroxide-treated cells cultured in low but not high glucose compared with untreated controls (Figure 6b). Protein levels of HIF1*α* were higher in peroxide-treated cells cultured in either high or low glucose compared with non-peroxide-treated controls (Figure 6c). However, using a HIF luciferase reporter, we found that HIF activity was only significantly higher in peroxide-treated cells cultured in low but not high glucose (Figure 6d). To confirm that HIF1*α* was transcriptionally active under these conditions, we infected tenocytes with an adenoviral construct carrying a *HIF1α*-targeting silencing RNA (siRNA) sequence adsiHIF1*α* (adenoviral vector bearing silencing RNA targeting hypoxia-inducible factor 1*α*). We found that RNA levels of known HIF target genes were significantly higher in peroxide-treated adGFP controls but not in peroxide-treated adsiHIF1*α* cells (Supplementary Figure).

To determine the involvement of p38*α*, we treated cells cultured in low glucose with peroxide and a p38 inhibitor. We found that HIF reporter activity was significantly lower (Figure 6e), but FOXO reporter activity significantly higher (Figure 6f), in peroxide-treated cells cultured in low glucose treated with the p38 inhibitor compared with non-inhibitor-treated cells. Both HIF1*α* and FOXO1 directly regulate transcription of superoxide dismutase 2 (SOD2).^30, 31^ We found that RNA levels of *SOD2* were significantly higher in peroxide-treated cells cultured in low glucose compared with untreated controls (Figures 6g and h). Knockdown of FOXO1 using RNAi had no effect on *SOD2* expression (Figure 6g); however, RNA levels of *SOD2* were significantly lower in cells in which HIF1*α* expression had been knocked down (Figure 6h), providing further support for the notion that HIF1*α* rather than FOXO1 was transcriptionally active under these conditions.

Having established that extracellular glucose levels determine whether HIF1*α* or FOXO1 is transcriptionally active following peroxide treatment, we wanted to determine the glucose concentration at which this switch occurred. We found that the activity of the HIF reporter was significantly higher than untreated controls in peroxide-treated cells cultured in 6 mM glucose, but not at higher glucose concentrations (Figure 6i). Conversely, activity of the FOXO reporter was only significantly higher than untreated controls in peroxide-treated cells cultured in ≥7 mM glucose (Figure 6i).

### HIF1*α* promotes tenocyte differentiation by upregulating expression of sox 9 and scleraxis

**Figure 7:**
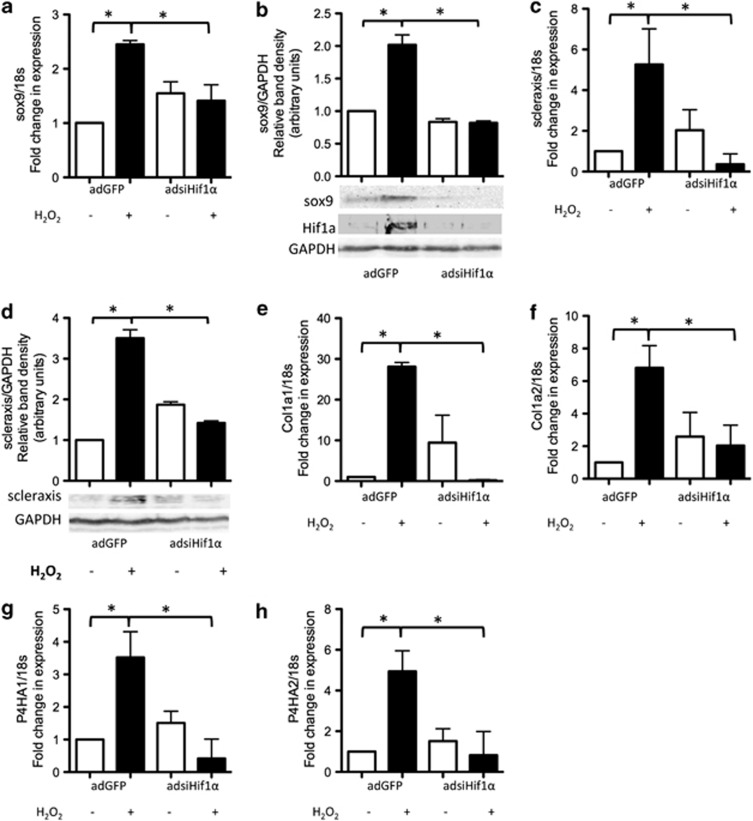
HIF1*α* upregulates tenocytes marker expression. An adenoviral vector bearing siRNA targeting *HIF1α* was used to knock down HIF1*α* expression in primary human tenocytes. A GFP-bearing adenoviral vector was used as a control. (**a**) RNA and (**b**) protein levels of sox9 were higher in adGFP-infected peroxide-treated cells cultured in low glucose compared with either adGFP-infected non-peroxide-treated cells or adsiHIF1*α*-infected peroxide-treated cells. Similarly, (**c**) RNA and (**d**) protein levels of scleraxis were higher in adGFP-infected peroxide-treated cells cultured in low glucose compared with either adGFP-infected non-peroxide-treated cells or adsiHIF1*α*-infected peroxide-treated cells. RNA levels of (**e**) *col1a1* and (**f**) *col1a2* were also significantly higher in adGFP-infected peroxide-treated cells cultured in low glucose compared with either adGFP-infected non-peroxide-treated cells or adsiHIF1*α*-infected peroxide-treated cells. RNA levels of the collagen prolylhydroxylases (**g**) *P4HA1* and (**h**) *P4HA2* were also significantly higher in adGFP-infected peroxide-treated cells cultured in low glucose compared with either adGFP-infected non-peroxide-treated cells or adsiHIF1*α*-infected peroxide-treated cells. A *P*-value of ≤0.05 was considered statistically significant. Results are expressed as mean±S.D. All experiments were performed on cells isolated from at least three different patients. Images shown are representative of those obtained for all patients

Finally, we wanted to determine what effect increased HIF1*α* activity had on cell fate in oxidative stress-exposed cells in low glucose. We found that RNA (Figure 7a) and protein (Figure 7b) levels of sox9, a critical regulator of both chondrocyte and tenocyte differentiation^32^ and a known HIF1 target gene,^33^ were significantly higher in peroxide-treated adGFP-infected cells cultured in low glucose compared with untreated controls. No change in either RNA (Figure 7a) or protein (Figure 7b) levels of sox9 occurred in peroxide-treated cells infected with adsiHIF1*α*. Similar to sox9, we found that RNA (Figure 7c) and protein (Figure 7d) levels of scleraxis, a key determinant of tenocyte differentiation,^32^ were significantly higher in peroxide-treated adGFP-infected cells cultured in low glucose, but not in peroxide-treated cells infected with adsiHIF1*α*.

A discriminating feature of the tenocyte, as opposed to the chondrocyte phenotype, is the type of collagen produced; tenocytes produce type 1 collagen, whereas chondrocytes produce type 2 collagen. Expression of *col1a1* and *col1a2*, the gene products of which form type 1 collagen, was significantly higher in peroxide-treated adGFP-infected but not adsiHIF1*α*-infected cells cultured in low glucose compared with controls (Figures 7e and f). In contrast, *col2a1* expression was not detectable in peroxide-treated cells cultured in low glucose for up to 72 h (data not shown).

Collagen prolylhydroxylases are enzymes essential for extracellular collagen deposition. We found that RNA levels of the prolyl-4-hydroxylases *P4HA1* (Figure 7g) and *P4HA2* (Figure 7h) were significantly higher in peroxide-treated adGFP-infected cells, but not in peroxide-treated adsiHIF1*α*-infected cells compared with untreated controls. These data indicate that HIF1*α* activity may promote both the synthesis as well as extracellular deposition of type 1 collagen.

## Discussion

In healthy individuals, blood glucose is tightly maintained within the range of 4–7 mM. In diabetics, levels can rise considerably higher. Serious health consequences can occur when levels exceed 13 mM, and concentrations of ∼33 mM are often lethal.^34^ In the present study, peroxide treatment of tenocytes cultured in standard high-glucose medium (17.5 mM, equivalent to a hyperglycemic blood glucose concentration) led to a significant increase in apoptosis. However, in cells cultured in 5 mM glucose (a normoglycemic blood glucose concentration), exposure to the same level of oxidative stress resulted in no loss of cell viability and instead led to increased synthesis of type 1 collagen, the major protein component of tendon.

We found that the apoptosis induction in peroxide-treated cells cultured in high glucose was dependent on the presence of both FOXO1 and p53, and was at least partly due to a cooperative effect of the two transcription factors on regulation of bim expression. FOXO1 is known to directly promote bim transcription,^23^ whereas p53 has previously been shown to inhibit expression of miR17-92,^26^ a cluster of miRNAs that includes the bim repressor miR17-5p.^27^ Results from our study indicate that p53-mediated inhibition of miR17-92, and consequently of miR17-5p, contributes to the upregulation of bim RNA levels in oxidative stress-exposed cells cultured in high glucose. Our data suggest that, in order for bim RNA levels to accumulate, both increased transcription (mediated by FOXO1) as well as prevention of bim RNA degradation (mediated by p53) are required. FOXOs and p53 are known to cooperatively mediate apoptosis by a variety of mechanisms.^35, 36, 37^ They are also known to reciprocally regulate each other's activation.^35^ The extent of cross-talk that can occur between p53 and FOXO1 is exemplified in the present study by the almost complete protection against apoptosis induction when expression of either of these transcription factors was ablated. It should be noted, however, that FOXO-mediated cell death is not always dependent on p53.^38, 39^ FOXOs and p53 can form partnerships with a number of other transcription factors allowing for context-specific regulation of their function.^39^

The increase in levels of acetylated p53 in peroxide-treated cells in high glucose in the present study was at least partially due to reduced levels of sirtuin 3, a p53 deacetylase and regulator of p53 activity.^29^ We found that peroxide treatment under high-glucose conditions resulted in miR28-5p-mediated inhibition of sirtuin 3, identifying sirtuin 3 as a novel target of this miRNA. Recently, nuclear factor (erythroid-derived 2)-like 2, an oxidative stress-induced transcription factor involved in antioxidant defence,^40^ was also identified as a target of miR28.^41^ Increased miR28 expression by oxidative stress under high glucose conditions may therefore have far-reaching consequences leading to impaired antioxidant defence as well as activation of proapoptotic pathways. Reduced antioxidant defence is likely to further promote p53 activation and may have contributed to the increase in phosphorylation of p53 we observed in peroxide-treated cells under high glucose.

The lack of FOXO1 transcriptional activity in peroxide-treated cells under low glucose conditions in the present study was surprising given that FOXO1 could be detected in the cell nucleus and that FOXO1 was also acetylated. Acetylation of FOXOs is essential for their binding to the FOXO response element in target genes such as bim^23^ and hence is usually an indicator of FOXO activation. However, FOXOs are subject to regulation by a number of different enzymes, including the p38 MAPK that directly phosphorylates FOXOs.^42^ Under low glucose conditions, we found that peroxide treatment led to increased activation of p38*α*, which in turn inhibited FOXO1 transcriptional activity. This is consistent with previous reports describing inhibition of FOXO1-dependent transcription by p38,^14, 42^ but in contrast to studies that have shown a stimulatory effect of p38 on nuclear localization and subsequent activation of FOXO3a,^43, 44^ suggesting p38 may differentially regulate different members of the FOXO family. Interestingly, Chiacchiera *et al.*^14^ found that inhibition of p38*α* initiated a switch from HIF1*α*- to FOXO-dependent transcription in colorectal cancer cells.^14^ In our study, although protein levels of HIF1*α* were higher in peroxide-treated cells compared with untreated controls regardless of extracellular glucose, HIF1*α* was only transcriptionally active in cells cultured in low glucose. In keeping with the findings of Chiacchiera *et al.*,^14^ this activation was found to be dependent on p38*α* and is likely a result of direct phosphorylation of Hif1*α* by p38, a mechanism known to promote Hif1*α* transcriptional activity.^45^

Activation of p38 can lead to either prosurvival or proapoptotic effects depending on the stimulus. For instance, under high-glucose conditions, p38 activation contributes to the induction of apoptosis.^46^ However, activation of p38*α* by oxidative stress promotes cell survival.^47^ The difference in outcome of p38 signalling depends on both the isoform activated as well as the presence of other interacting regulatory pathways.^48^ In the present study, we observed increased p38*α* activation only in peroxide-treated cells in low glucose, not high glucose. This raises the possibility that high glucose prevents the normal oxidative stress-induced (and hence prosurvival) activation of p38.

Finally, we found that peroxide treatment of tenocytes cultured in low glucose led to increased expression of both col1a1 and col1a2, the gene products of which form the type I collagen heterotrimer. This increase in type I collagen synthesis was dependent on HIF1*α,* and was likely due to HIF1*α*-mediated promotion of scleraxis expression. Scleraxis is a marker of the tenocyte lineage and is itself a transcription factor, which directly regulates expression of col1a1 and col1a2.^49, 50^ To our knowledge, regulation of scleraxis expression by HIF1 is a novel finding and may have important implications for the understanding of tenocyte differentiation and tendon development.

**Figure 8:**
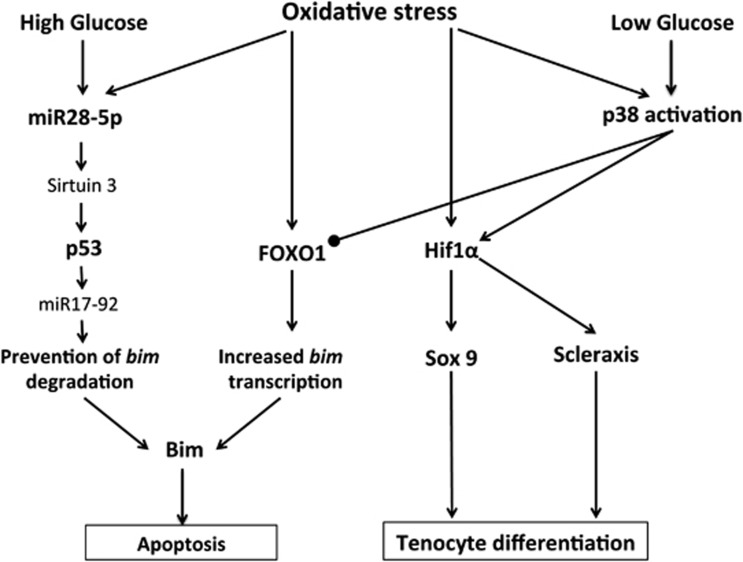
Proposed mechanism for the differential response of tenocytes to oxidative stress under different extracellular glucose concentrations. Oxidative stress results in upregulation of both FOXO1 as well as HIF1*α*. Under high-glucose conditions, miR28-5p levels are also upregulated, particularly in oxidative stress-exposed cells. miR28-5p directly inhibits expression of the p53 deacetylase sirtuin 3, allowing accumulation of acetylated p53. FOXO1 promotes transcription of *bim*, the gene product of which is a proapoptotic protein. p53 inhibits expression of miR17-92, a cluster of miRNAs including the bim repressor miR17-5p. Downregulation of miR17-92 by p53 coupled with increased *bim* transcription by FOXO1 allows accumulation of bim RNA levels and increased bim-mediated apoptosis. Under low-glucose conditions, however, the miR28-5p–sirt3-p53 pathway is not stimulated. Instead, p38 MAPK is activated and acts on both FOXO1 and HIF1*α*, resulting in the inhibition of FOXO1 transcriptional activity and activation of HIF1*α*. HIF1*α* promotes the expression of *sox9* and *scleraxis*, two genes whose products are essential for tenocyte differentiation

Results from the present study suggest an intriguing scenario in which oxidative stress results in indiscriminate upregulation of both anabolic and proapoptotic factors. Which of the two pathways prevails depends on extracellular glucose levels (Figure 8). Interestingly, we found that the switch in activation of these two divergent pathways occurred at an extracellular glucose level of 7 mM, a concentration equivalent to the maximal normal blood glucose level. These results may imply that elevated blood glucose levels even in the non-diabetic range could predispose to oxidative stress-induced apoptosis at the expense of normal tissue maintenance and repair, leading to tissue degeneration.

## Materials and Methods

### Materials

Primary antibodies used in this study were as follows: sirtuin 1 clone E54, Novus Biologicals, Cambridge, UK; p53 clone DO-1, Life Technologies, Paisley, UK; FOXO3a clone 75D8, FKHR (FOXO1) clone EP927Y, phospho-FOXO1 (Ser256) (cat. no. 9461), acetylated p53 (K382) clone EPR358(2), sirtuin 3 clone C73E3, phospho-p53 (cat. no. 9284P); phospho-Akt (S473) (clone 193H12 and pan Akt (cat. no. 9272), all from Cell Signaling Technologies, Danvers, MA, USA; Bim (cat. no. 202000), Merck KGaA, Darmstadt, Germany; HIF1*α* (cat. no. 610959), BD Biosciences, Oxford, UK; acetyl-FOXO1 (K259, K262, K271) (cat. no. sc-49437) Santa Cruz Biotechnology; scleraxis (cat. no. ab58655), Abcam, Cambridge, UK; sox9 (cat. no. HPA001758) Sigma, Poole, UK; phospho-p38 (T180/Y182) (cat. no. AF869) and pan p38*α* (cat no. AF8691) both from R&D Systems, Abingdon, UK. Cells-to-cDNA lysis buffer, DNase I and DNase buffer were purchased from Ambion, Austin, TX, USA. Random primers, reverse transcriptase (RT) buffer, MMLV reverse transcriptase, SYBR green and Taqman Master Mixes were purchased from Life Technologies. Quantitect primer assays for sirtuin 1, sirtuin 3, puma, lactate dehydrogenase, miR17-92 and noxa were purchased from Qiagen Ltd, Crawley, UK. Taqman primer/probe sets for bim, vascular endothelial growth factor-A, miR17-5p and miR28-5p were purchased from Life Technologies. Mimic and inhibitors for miR17-5p and miR28-5p as well as the respective controls were purchased from Life Technologies. The sirtuin 3 adenoviral vector (adSirt3) was purchased from Vector Biolabs, Philadelphia, PA, USA. The siHIF1*α* adenoviral vector (adsiHIF1*α*) was custom-made by ABM Good, Richmond, BC, Canada. The GFP adenoviral vector (adGFP) was a kind gift from Dr Jillian Cornish, University of Auckland, NZ. Fetal bovine serum was purchased from Biosera, Boussens, France. PFT*α* and SB202190 were purchased from Sigma. Unless otherwise stated, all other chemicals were purchased from Sigma and were of the highest purity available.

### Cell culture

Tendon tissue was obtained from the Oxford Musculoskeletal BioBank and was collected with informed donor consent in full compliance with national and institutional ethical requirements, the United Kingdom Human Tissue Act, and the Declaration of Helsinki. Human tenocytes were obtained by explant culture of hamstring tendon used in repair of ruptured anterior cruciate ligament as previously described.^51^ Previous studies demonstrate that primary tenocytes remain phenotypically stable until passage 5 when passaged subconfluence.^52^ In the following experiments, cells were passaged at 70% confluence and used up until passage 3. Cells were routinely cultured in DMEM/F12 (containing 17.5 mM glucose ‘high glucose', cat. no. BE12–719F, Lonza Group, Basel, Switzerland) supplemented with 5% FBS without antibiotics. Low glucose (5 mM) DMEM-F12 was made by mixing Hams-F12 (cat. no. BE12–615F, Lonza Group) with glucose-free DMEM (cat. no. E15–079, PAA Laboratories (now GE Healthcare, Little Chalfont, UK)) in a 50 : 50 ratio. The glucose-free DMEM was supplemented with 5 mM Glutamax (Life Technologies), 15 mM HEPES and 5 mM sodium pyruvate (both from PAA Laboratories) to ensure the formulation was identical to that of high-glucose DMEM-F12. The osmolarity range as specified by the manufacturer of glucose-free DMEM, Hams-F12 and DMEM-F12 was the same (286–356 mOsm).

### RNAi-mediated gene silencing

Cells were cultured for 18 h in serum-free DMEM-F12 with lipofectamine RNAimax (Life Technologies) (1.64 *μ*l/ml) and 36 pM siRNA (nontargeting control catalogue number D-001810-01-05, ThermoScientific, Rockford, IL, USA; FOXO1, p53 or puma, catalogue numbers SI02781415, SI02655170 and SI02655520 respectively, all from Qiagen Ltd) or bim (catalogue number 4390824, Ambion)). Lipofectamine-containing media was subsequently removed and replaced with standard growth media (DMEM-F12 containing 5% FBS) for further 24 h before treating as appropriate for individual experiments. The success of gene knockdown was confirmed by western blotting 48 h after transfection.

### Adenoviral-mediated gene transduction

Cells were plates at 50 000/ml and allowed to adhere overnight. Tenocytes were infected with the appropriate adenoviral vector at a multiplicity of infection of 35 using Xtremegene HP (Roche Diagnostics Ltd, Burgess Hill, UK) following the manufacturer's instructions. The sequence of the HIF1*α*-targeting siRNA duplex in the adsiHif1*α* construct was 5′-CUGAUGACCAGCAACUUGAdTdT-3′ and 5′-UCAAGUUGCUGGUCAUCAGdTdT-3′ as previously validated.^53^

### Western blotting

Except for HIF1*α* blots, cells were sonicated in standard lysis buffer containing protease and phosphatase inhibitors. For HIF1*α* blots, cells were sonicated in HIF lysis buffer (10% glycerol, 1% Tris-HCl pH 6.8 (1 M), 0.5% DTT (1 M), 0.01% SDS, 0.01% leupeptin (5 mg/ml), 0.02% pepstatin (10 mg/ml), and 39% urea (8 mM)). Proteins were quantified using the Pierce 660 protein determination assay (non-HIF samples, ThermoScientific) or the Bradford BCA assay (HIF lysates, ThermoScientific) and lysates diluted as appropriate to ensure equal loading of total protein. Western blots were carried out according to standard protocols^51^ and proteins visualised using Pierce WestDura detection reagents (ThermoScientific) using a Chemi Doc-It imaging system with Biochemi HR camera (UVP, Upland, CA, USA).

### Real-time RT-PCR

cDNA was prepared using a cells-to-cDNA kit as per the manufacturer's instructions (Ambion). Samples without reverse transcriptase and no-template controls served as negative controls. Real-time qPCR reactions were performed using a ViiA7 Real Time PCR System (Life Technologies). All samples were run in duplicate with a coefficient of variation between duplicates of <1.0 cycle. Analysis was carried out using the delta-delta cT method.^54^

### HIF and FOXO reporter assays

Tenocytes were transfected with pHRG-TK Renilla luciferase (Promega, Madison, WI, USA) and either PGK hypoxia-inducible factor response element-firefly luciferase (a gift from Professor Adrian Harris, University of Oxford, UK) or forkhead response element-luciferase (created by Michael Greenberg and described in Brunet *et al.*^7^, Addgene plasmid 1789, Addgene, Cambridge, MA, USA) plasmids using lipofectamine 2000 (Life Technologies). Luciferase activity was determined using the Dual-glo Luciferase assay (Promega) 8 h following treatments.

### Transfection of miR mimics and inhibitors

Tenocytes were plated at 10 000 cells/well in 96-well plates and allowed to adhere overnight. The next day, cells were transfected with either 50 nM of miRNA inhibitor or inhibitor control or 30 nM of miRNA mimic or mimic control (all from Life Technologies) using lipofectamine 2000 (Life Technologies). After 6 h, lipofectamine-containing media was removed and cells were cultured in standard growth media containing 5% FBS for 48 h. Cells were then media changed to either high- or low-glucose media with or without peroxide as appropriate for individual experiments and cultured for a further 8 h before collection of RNA for assessment of changes in gene expression.

### 3′-UTR reporter assay

Cells were seeded at 10 000/well in a 96-well plate and allowed to adhere overnight. Cells were then transfected with 100 ng of either negative control 3′-UTR (cat. no. CmiT000001-MT05) or Sirt3 3′UTR (cat no. HmiT006079-MT05) GLuc-SEAP vector (GeneCopoeia, Rockville, MD, USA) with 30 nM of either miR28-5p mimic or mimic control using lipofectamine 2000 (all from Life Technologies). Following 6 h, media was changed to standard culture media containing 5% FBS for further 18 h. Gaussia luciferase and secreted alkaline phosphatase activities were measured using a Secrete Pair TM Dual Luminescence Assay Kit following the manufacturer's instructions (GeneCopoeia). The Sirt3 3′-UTR reporter contained the GLuc gene under the control of the SV40 promoter. The 3′-UTR of sirtuin 3 was inserted downstream of GLuc, thus leading to the formation of a chimeric mRNA. A secreted alkaline phosphatase (SEAP) reporter driven by the CMV promoter cloned into the same vector served as an internal control for transfection efficiency.

### Immunocytochemistry

Tenocytes were plated at a density of 50 000 cells/ml on coverglasses in 24-well plates and allowed to adhere overnight. Media was then changed to either high or low glucose with or without peroxide for a further 8 h. Cells were then fixed with 10% formaldehyde for 30 min. Following washing, cells were permeabilised with 0.5% triton-X in PBS for 5 min. Non-specific binding sites were blocked by incubating in 3% horse serum for 1 h at room temperature before incubation in FOXO1 primary antibody (diluted 1 : 50) for 18 h at 4 °C in a humidified chamber. Following thorough washing, cells were incubated with Dylight-488 anti-rabbit secondary antibody (Thermo-Fisher Scientific Ltd, Rockford, IL, USA; diluted 1 : 100 with PBS) for 45 min at room temperature, and then 4′6-diamidino-2-phenylindole dichloride (DAPI) for a further 30 min at room temperature. Cells were then thoroughly washed with ddH_2_O before mounting in VectaShield (Vector Laboratories Inc, Burlingame, CA, USA) and imaging on an Olympus BX40 microscope using an Olympus U-CMAD3 camera (Olympus, Southend-on-Sea, UK). IgG and secondary antibody controls were used to verify the specificity of the primary antibody.

### Statistical analysis

All experiments were repeated at least three times using different tissue donors for each experimental replicate. Results were analysed by one-way ANOVA with *post hoc* Tukey testing or by *t*-test if only two conditions were being tested. All data were analysed using Prism 5.0 b (GraphPad Software, La Jolla, CA, USA). *P-*value ≤0.05 was considered statistically significant. Results are expressed as mean±S.D.

## Abbreviations

FOXO: forkhead box O
HIF: hypoxia-inducible factor
MAPK: mitogen-activated protein kinase
ROS: reactive oxygen species
PI3K: phosphatidylinositol-3-kinase
PKB: protein kinase B
RNAi: RNA interference
miR: micro RNA
PFT*α*: pifithrin-*α*
sirt3: sirtuin 3
adSirt3: adenoviral vector bearing a sirtuin 3 construct
GFP: green fluorescent protein
adGFP: adenoviral vector bearing a green fluorescent protein construct
adsiHIF1*α*: adenoviral vector bearing silencing RNA targeting hypoxia-inducible factor 1*α*
siRNA: silencing RNA
SOD2: superoxide dismutase 2
P4HA: prolyl-4-hydroxylase
DAPI: 4′6-diamidino-2-phenylindole dichloride

## References

[bib1] Sauer H Wartenberg M Hescheler J Reactive oxygen species as intracellular messengers during cell growth and differentiation Cell Physiol Biochem 2001 11 173 186 11509825 10.1159/000047804

[bib2] Li D Xie G Wang E Reactive oxygen species: the 2-edged sword of osteoarthritis Am J Med Sci 2012 344 486 490 22885622 10.1097/MAJ.0b013e3182579dc6

[bib3] Ames B Shigenaga M Hagen T Oxidants, antioxidants and the degenerative diseases of aging Proc Natl Acad Sci 1993 90 7915 7922 8367443 10.1073/pnas.90.17.7915 PMC47258

[bib4] Gambino V De Michele G Venezia O Migliaccio P Dall'Olio V Bernard L Oxidative stress activates a specific p53 transcriptional response that regulates cellular senescence and aging Aging Cell 2013 12 435 445 23448364 10.1111/acel.12060 PMC3709138

[bib5] Kops G Dansen TB Polderman PE Saarloos I Wirtz KWA Coffer PJ Forkhead transcription factor FOXO3a protects quiescent cells from oxidative stress Nature 2002 419 316 321 12239572 10.1038/nature01036

[bib6] Tothova Z Kollipara R Huntly B Lee B Castrillon D Cullen D FoxOs are critical mediators of hematopoietic stem cell resistance to physiologic oxidative stress Cell 2007 128 325 339 17254970 10.1016/j.cell.2007.01.003

[bib7] Brunet A Bonni A Zigmond MJ Lin MZ Juo P Hu LS Akt promotes cell survival by phosphorylating and inhibiting a forkhead transcription factor Cell 1999 96 857 868 10102273 10.1016/s0092-8674(00)80595-4

[bib8] Lowe S Schmitt E Smith S Osborne B Jacks T p53 is required for radiation-induced apoptosis in mouse thymocytes Nature 1993 362 847 849 8479522 10.1038/362847a0

[bib9] Cohen H Miller CM Bitterman KJ Wall N Hekking B Kessler B Calorie restriction promotes mammalian cell survival by inducing the SIRT1 deacetylase Science 2004 305 390 392 15205477 10.1126/science.1099196

[bib10] Igarashi M Wakasaki H Takahara N Ishii H Jiang Z Yamauchi T Glucose or diabetes activates p38 mitogen-activated protein kinase via different pathways J Clin Invest 1999 103 185 195 9916130 10.1172/JCI3326 PMC407875

[bib11] Ogawar Y Kishishita S Obata T Isazawa Y Suzuki T Tanaka K Akt enhances Mdm2-mediated ubiquitination and degradaton of p53 J Biol Chem 2002 277 21843 21850 11923280 10.1074/jbc.M109745200

[bib12] Agani F Jiang B Oxygen-independent regulation of Hif-1: novel involvement of PI3K/AKT/mTOR pathway in cancer Curr Cancer Drug Targets 2013 13 245 251 23297826 10.2174/1568009611313030003

[bib13] Hubbi M Hu H Kshitiz Gilkes D Semenza G Sirtuin-7 inhibits the activity of hypoxia-inducible factors J Biol Chem 2013 288 20768 20775 23750001 10.1074/jbc.M113.476903 PMC3774348

[bib14] Chiacchiera F Matrone A Ferrari E Ingravallo G Lo Sasso G Murzilli S p38*α* blockade inhibits colorectal cancer growth *in vivo* by inducing a switch from Hif1*α*- to FoxO-dependent transcription Cell Death Differ 2009 16 1203 1214 19343039 10.1038/cdd.2009.36

[bib15] Pagé E Chan D Giaccia A Levine M Richard D Hypoxia-inducible factor 1*α* stabilization in nonhypoxic conditions: role of oxidation and intracellular ascorbate depletion Mol Biol Cell 2008 19 86 94 17942596 10.1091/mbc.E07-06-0612 PMC2174200

[bib16] Basu R Hibchak S Hayashida T Runyan C Schumacker P Schnaper H Interdependence of Hif-1 alpha and TGF-beta/Smad3 signaling in normoxic and hypoxic renal epithelial cell collagen expression Am J Physiol Renal Physiol 2011 300 F898 F905 21209004 10.1152/ajprenal.00335.2010 PMC3075002

[bib17] Gilkes D Bajpai S Chaturvedi P Wirtz D Semenza G Hypoxia-inducible factor 1 (Hif-1) promotes extracellular matrix remodeing under hypoxic conditions by inducing P4HA1, P4HA2 and PLOD2 expression in fibroblasts J Biol Chem 2013 288 10819 10829 23423382 10.1074/jbc.M112.442939 PMC3624462

[bib18] Umegaki H Neurodegeneration in diabetes mellitus Adv Exp Med Biol 2012 724 258 265 22411248 10.1007/978-1-4614-0653-2_19

[bib19] Peterson Kim R Edelma S Kim D Musculoskeletal complications of diabetes mellitus Clin Diabetes 2001 19 132 135

[bib20] Sukenik S Weitzman S Buskila D Eyal A Gross J Horowitz J Limited joint mobility and other rheumatological manifestations in diabetic patients Diabetes Metab 1987 13 187 192 3609420

[bib21] Rechardt M Shiri R Karppinen J Jula A Heliovaara M Viikari-Juntura E Lifestyle and metabolic factors in relation to shoulder pain and rotator cuff tendinitis: a population-based study BMC Musculoskeletal Dis 2010 11 11 10.1186/1471-2474-11-165 PMC3161397 20646281

[bib22] Carson D Ribeiro J Apoptosis and disease Lancet 1993 341 1251 1254 8098400 10.1016/0140-6736(93)91154-e

[bib23] Yang Y Zhao Y Liao W Yang J Wu L Zheng Z Acetylation of FoxO1 activates Bim expression to induce apoptosis in response to histone deacetylase inhibitor depsipeptide treatment Neoplasia 2009 11 313 324 19308286 10.1593/neo.81358 PMC2657887

[bib24] Polyak K Xia Y Zweier J Kinzler K Vogelstein B A model for p53-induced apoptosis Nature 1997 389 300 305 9305847 10.1038/38525

[bib25] Chuang PY Yu Q Fang W Uribarri J He JC Advanced glycation endproducts induce podocyte apoptosis by activation of the FOXO4 transcription factor Kidney Int 2007 72 965 976 17667983 10.1038/sj.ki.5002456 PMC3191877

[bib26] Yan HL Xue G Mei Q Wang YZ Ding FX Liu MF Repression of the miR-17-92 cluster by p53 has an important function in hypoxia-induced apoptosis EMBO J 2009 28 2719 2732 19696742 10.1038/emboj.2009.214 PMC2750010

[bib27] Fontana L Fiori M Albini S Cifaldi L Giovinazzi S Forloni M Antagomir-17-5p abolishes the growth of therapy-resistant neuroblastoma through p21 and bim Plos One 2008 3 e2236 18493594 10.1371/journal.pone.0002236 PMC2375057

[bib28] Vaziri H Dessain SK Eagon EN Imai SI Frye RA Pandita TK hSIR2(SIRT1) functions as an NAD-dependent p53 deacetylase Cell 2001 107 149 159 11672523 10.1016/s0092-8674(01)00527-x

[bib29] Li SD Banck M Mujtaba S Zhou MM Sugrue MM Walsh MJ p53-induced growth arrest is regulated by the mitochondrial SirT3 deacetylase Plos One 2010 5 e10486 20463968 10.1371/journal.pone.0010486 PMC2864751

[bib30] Colla S Storti P Donofrio G Todoerti K Bolzoni M Lazzaretti M Low bone marrow oxygen tension and hypoxia-inducible factor-1*α* overexpression characterize patients with multile myeloma: role on the transcriptional and proangiogenic profiles of CD138+ cells Leukemia 2010 24 1967 1970 20811474 10.1038/leu.2010.193

[bib31] Calnan D Brunet A The FoxO code Oncogene 2008 27 2276 2288 18391970 10.1038/onc.2008.21

[bib32] Siugimoto Y Takimoto A Akiyama H Kist R Scherer G Nakamura T Scx(+)/Sox9(+) progenitors contribute to the establishment of the junction between cartilage and tendon/ligament Development 2013 140 2280 2288 23615282 10.1242/dev.096354

[bib33] Amarilllo R Viukov S Sharir A WEshkar-Oren I Johnson R Zelzer E Hif1alpha regulation of sox9 is necessary to maintain differentiation of hypoxic prechondrogenic cells during early skeletogenesis Development 2007 134 3917 3928 17913788 10.1242/dev.008441

[bib34] McNaughton C Self W Slovis C Diabetes in the emergency department: acute care of diabetes patients Clin Diabetes 2011 29 51 59

[bib35] Renault V Thekkat P Hoang K White J Brady C Kenzelmann Broz D The pro-longevity gene FOXO3 is a direct target of the p53 tumor suppressor Oncogene 2011 1 15 10.1038/onc.2011.35 PMC3136551 21423206

[bib36] You H Yamamoto K Mak TW Regulation of transactivation-independent proapoptotic activity of p53 by FOXO3a Proc Nat Acad Sci 2006 103 9051 9056 16757565 10.1073/pnas.0600889103 PMC1482564

[bib37] Bouchard C Lee S Paulus-Hock V Loddenkemper C Eilers M Schmitt C FoxO transcription factors suppress Myc-driven lymphomagenesis via direct actiavtion of Arf Genes Develop 2007 21 2775 2787 17974917 10.1101/gad.453107 PMC2045131

[bib38] Yang J Xia W Hu M Inonizing radiation activates expression of FOXO3a, Fas ligand and bim and induces apoptosis Int J Oncol 2006 29 643 648 16865280 PMC2632978

[bib39] Eijkelenboom A Burgering M FOXOs: signalling integrators for homeostasis maintenance Nature Rev Mol Cell Biol 2013 14 83 97 23325358 10.1038/nrm3507

[bib40] Leiser SF Miller RA Nrf2 signaling, a mechanism for cellular stress resistance in long-lived mice Mol Cell Biol 2010 30 871 884 19933842 10.1128/MCB.01145-09 PMC2812245

[bib41] Yang M Yao Y WEades G Zhang Y Zhou Q MiR-28 regulates Nrf2 expression through a Keap1-independent mechanism Breast Cancer Res Treat 2011 129 983 991 21638050 10.1007/s10549-011-1604-1 PMC3752913

[bib42] Asada S Daitoku H Matsuzaki H Saito T Sudo T Mukai H Mitogen-activated protein kinases Erk and p38 phosphorylate and regulate FOXO1 Cell Signal 2007 19 519 527 17113751 10.1016/j.cellsig.2006.08.015

[bib43] Ho K McGuire V Koo C Muir K de Olano N Maifoshie E Phosphorylation of FOXO3a on Ser-7 by p38 promotes its nuclear localisation in response to doxorubicin J Biol Chem 2012 287 1545 1555 22128155 10.1074/jbc.M111.284224 PMC3256863

[bib44] Clavel S Siffroi-Fernandez S Coldefy A Boulukos K Pisani D Dérijard B Regulation of intracellular localisation of FOXO3a by stress-activated ptotein kinase signaling pathways in skeletal muscle cells Mol Cell Biol 2010 30 470 480 19917721 10.1128/MCB.00666-09 PMC2798458

[bib45] Kwon S Song J Lee Y Signal pathway of hypoxia-inducible factor-1*α* phosphorylation and its interaction with von Hippel-Lindau tumour suppressor protein during ischemia in MiaPaCa-2 pancreatic cancer cells Clin Cancer Res 2005 11 7607 7613 16278378 10.1158/1078-0432.CCR-05-0981

[bib46] Nakagami H Morishita R Yamamoto K Yoshimura S Taniyama Y Aoki M Phosphorylation of p38 mitogen-activated protein kinase downstream of bax-caspase-3 pathway leads to cell death induced by high D-glucose in human endothelial cells Diabetes 2001 50 1472 1481 11375350 10.2337/diabetes.50.6.1472

[bib47] Gutierrez-Uzquiza Á Arechederra M Bragado P Aguirre-Ghiso J Porras A p38*α* mediates cell survival in response to oxidative stress via induction of antioxidant genes J Biol Chem 2012 287 2632 2642 22139847 10.1074/jbc.M111.323709 PMC3268422

[bib48] Nebreda A Porras A p38 MAP kinases: beyond the stress response Trends Biochem Sci 2000 25 257 260 10838561 10.1016/s0968-0004(00)01595-4

[bib49] Léjard V Brideau G Blais F Salingcarnboriboon R Wagner G Roehrl MHA Scleraxis and NFATc regulate the expression of the pro-alpha 1(I) collagen gene in tendon fibroblasts J Biol Chem 2007 282 17665 17675 17430895 10.1074/jbc.M610113200

[bib50] Espira L Lamoureux L Jones S Gerard R Dixon I Czubryt M The basic helix-loop-helix transcription factor scleraxis regulates fibroblast colagen synthesis J Mol Cell Cardiol 2009 47 188 195 19362560 10.1016/j.yjmcc.2009.03.024

[bib51] Poulsen R Carr A Hulley P Protection against glucocorticoid-induced damage in human tenocytes by modulation of ERK, Akt and forkhead signalling Endocrinology 2011 152 503 514 21209015 10.1210/en.2010-1087

[bib52] Yao L Bestwick CS Bestwick LA Maffulli N Aspden RM Phenotypic drift in human tenocyte culture Tissue Eng 2006 12 1843 1849 16889514 10.1089/ten.2006.12.1843

[bib53] Morten K Badder L Knowles HJ Differential regulation of Hif-mediated pathways increases mitochondrial metabolism and ATP production in hypoxic osteoclasts J Pathol 2013 229 755 764 23303559 10.1002/path.4159 PMC3618370

[bib54] Livak KJ Schmittgen TD Analysis of relative gene expression data using real-time quantitative PCR and the 2(-Delta Delta C(T)) Method Methods 2001 25 402 408 11846609 10.1006/meth.2001.1262

