# Supplementary figures and images for: Cell differentiation versus cell death: extracellular glucose is a key determinant of cell fate following oxidative stress exposure
Source: Cell Death Dis. 2014 Feb 20;5(2):e1074–. doi: 10.1038/cddis.2014.52 (PMC3944267; doi:10.1038/cddis.2014.52)

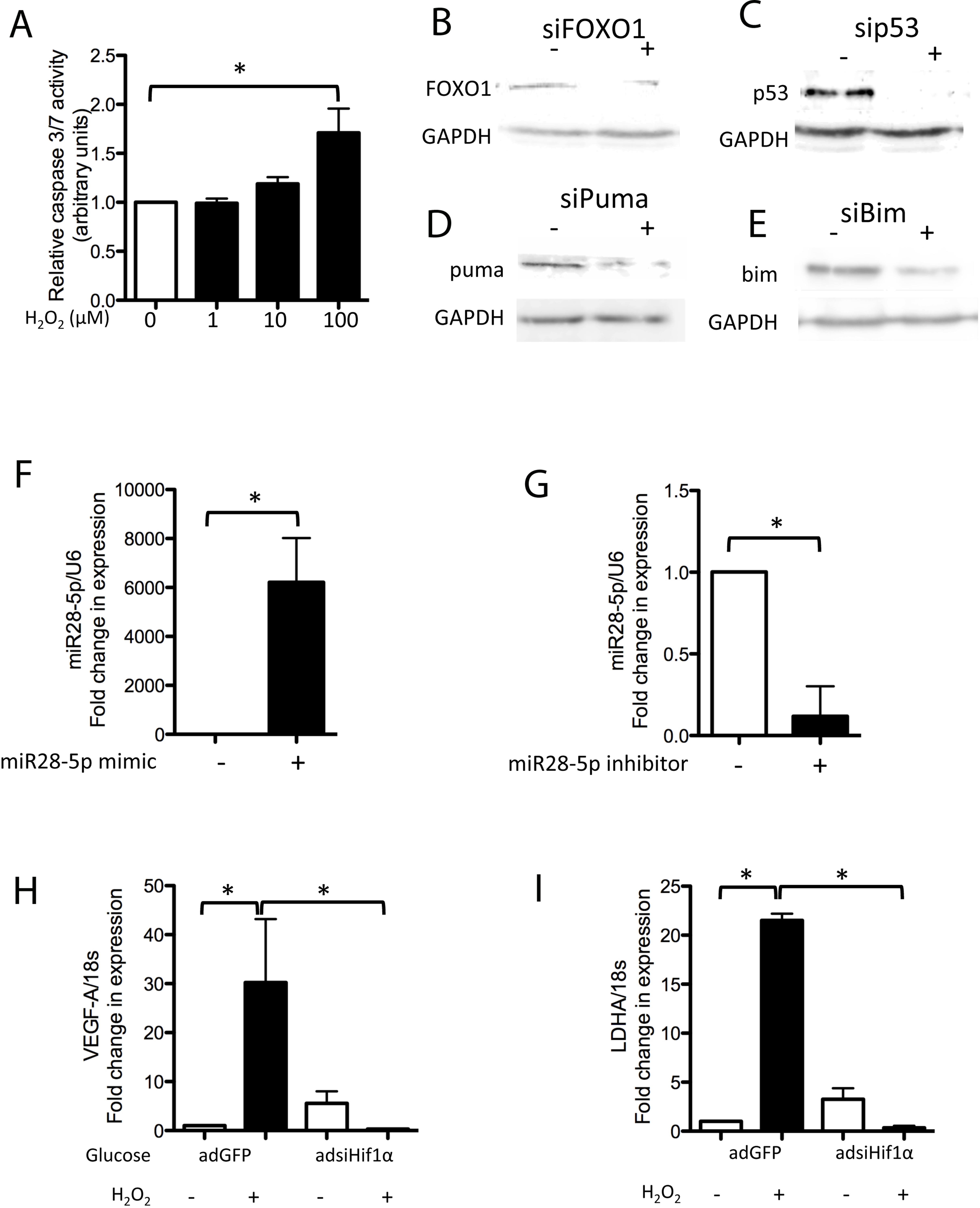

Supplement: Supplementary Figure [file cddis201452x1.tif]
