# Supplementary material for: Cell differentiation versus cell death: extracellular glucose is a key determinant of cell fate following oxidative stress exposure
Source: Cell Death Dis. 2014 Feb 20;5(2):e1074–. doi: 10.1038/cddis.2014.52 (PMC3944267; doi:10.1038/cddis.2014.52)
Supplement: Supplementary Figure Legend [file cddis201452x2.doc]

**Supplementary Figure**

**A** Level of apoptosis was significantly elevated in primary human tenocytes treated with 100μM (but not 10μM or 1μM) hydrogen peroxide for 18h compared to untreated controls. Western blots showing level of knockdown achieved at the protein level by transfection of primary human tenocytes with RNAi targeting **B** FOXO1, **C** p53, **D** puma and **E** bim. **F** Relative level of over-expression of miR28-5p obtained in primary human tenocytes 48h following transfection with a miR28-5p mimic compared to in cells transfected with a mimic control. **G** Levels of miR28-5p in primary human tenocytes 48h following transfection with a miR28-5p inhibitor compared to in cells transfected with an inhibitor control. RNA levels of known HIF1α-target genes **H** *VEGF-A* (vascular endothelial growth factor) and **I** *LDHA* (lactate dehydrogenase) were significantly higher in adGFP-infected peroxide-treated cells cultured in low glucose compared to untreated adGFP controls or compared to peroxide-treated cells infected with an adenoviral vector bearing a HIF1α-targetting siRNA duplex (adsiHIF1α). A p value of ≤0.05 was considered statistically significant. Results are expressed as mean ± standard deviation.
